# Haptic feedback as affective amplifier: enhanced fear perception affects cognitive performance and avoidance actions in VR height exposure

**DOI:** 10.3389/fpsyg.2025.1560157

**Published:** 2025-07-23

**Authors:** Lin Cong, Shan Cheng, Chaolin Teng, Kaiwen Xiong, Meiqing Huang, Jin Ma, Wendong Hu, Jianqi Wang, Weitao Dang, Kewei Sun, Taihui Zhang

**Affiliations:** ^1^School of Aerospace Medicine, The Fourth Military Medical University, Xi’an, China; ^2^School of Biomedical Engineering, The Fourth Military Medical University, Xi’an, China; ^3^Department of Military Medical Psychology, The Fourth Military Medical University, Xi’an, China

**Keywords:** fear, haptic feedback, avoidance behavior, EEG, virtual reality, threat perception

## Abstract

Haptic feedback serves as a potent affective amplifier in virtual reality (VR), intensifying threat perception to influence emotional intensity. This study investigated how haptic-enhanced fear stimuli (delivered through platform shaking during VR height exposure) impair cognitive performance and avoidance actions. Twenty male participants with self-reported fear of heights were subjected to four emotion-inducing conditions: neutrality, ground, stationary, and shaking, with each condition separated by 1 week. We have multimodal assessed of physiological (EEG, peripheral physiological arousal), behavioral (movement distance, eye movement), and cognitive performance (nine-light task accuracy rates and reaction time) during fear induction. Results revealed significant declines in task accuracy and prolonged reaction times during the shaking condition, indicating resource competition where threat processing impaired goal-directed motor execution. The decreased movement distance and increased pupil dilation indicated the initiation of an automatic defense response. Furthermore, physiological markers confirmed amplified this threat processing. The increased heart rate and elevated *β* band activity in EEG, indicating heightened cortical engagement with fear stimuli. The integration of physiological, behavioral, and subjective measures provides a mechanistic model for affective-motor competition, demonstrating how somatic threat cues translate into altered actions.

## 1 Introduction

Fear is an evolutionary emotion that serves as an adaptive response to environmental threats (Peleskova et al., 2023). However, fear, as a negative emotion, can disrupt perception, memory, and cognitive processes, impairing the ability to analyze and judge current situations, which may lead to disordered behavior (Reynolds and Askew, 2019). Critically, affective stimuli (especially threat-relevant cues) compete for attentional resources otherwise allocated to motor execution and inhibitory control (Pessoa, 2009; Battaglia et al., 2022). Advancements in neuroscience clarify that emotion dynamically interacts with cognition, especially executive functions governing motor preparedness and behavioral adaptation (Okon-Singer et al., 2015; Raschle et al., 2017; Reisenzein, 2019; Solms and Jurist, 2017). The dual competition model of cognitive-emotional interaction further posits that emotional intensity dictates whether affective stimuli enhance or impair motor execution, depending on their task relevance and demands on executive control (Pessoa, 2009). Low-intensity emotions are suggested to enhance sensory representation, thereby improving task-related performance (Senderecka, 2018; Sussman et al., 2013). Moderate emotional intensity tends to induce greater perceptual awareness and attention, promoting executive function (Kanske and Kotz, 2011; Nayak et al., 2019). In contrast, high-intensity emotions often impair task performance due to their demand for cognitive resources, as the brain prioritizes processing these stimuli (Hofstee et al., 2021; Jung et al., 2014). The methods and sources of stress used to induce fear significantly influence the emotional responses experienced by individuals. Current research on fear is extensive. However, due to the limitations in experimental paradigms and induction methods, studies frequently rely on conventional materials such as text, images, sounds, odors (Putkinen et al., 2021; Rohr et al., 2022; Vieillard et al., 2021; Zhang et al., 2019), or situational inductions through recollection and computer games (Ozawa, 2021; Villani et al., 2018; Zhang et al., 2014). Researchers have primarily concentrated on the mechanisms of emotional processing and the effects of emotional valence, frequently neglecting the influence of emotional arousal intensity (Sutherland and Mather, 2018). Traditional induction methods have demonstrated limitations in inducing high-intensity fear emotions, which restricts their research applicability. In recent years, researchers have increasingly employed virtual reality (VR) to investigate psychological phenomena. VR technology constructs virtual environment (VE) that provoke physiological and psychological responses, simulating high-risk and high-pressure situations while ensuring participant safety (Page and Coxon, 2016). This approach facilitates controlled and repeatable scenarios that accurately simulate everyday experiences, thus improving the ecological validity of psychological research (Bell et al., 2020).

Height is a common source of stress as epidemiological studies indicate that approximately one-third of individuals experience varying degrees of height intolerance (Teggi et al., 2019). Fear induced by height poses a significant risk to individuals, as many experience anxiety in high-altitude environments. Traditional methods for inducing acrophobia, such as physical exposure to climbing ladders or heights, are increasingly unsuitable for contemporary research due to limitations in venue conditions, safety, and procedural control (Teachman et al., 2008). Recent studies suggest that human psychological and physiological responses are comparable in both real and VE (Anglin et al., 2017; Browning et al., 2020). An individual’s reactions and interactions in different VE are influenced by various technical and psychological factors, with immersion and presence being the most significant. Immersion is an objective, technology-related factor of VE (Litleskare et al., 2020). In contrast, presence denotes the subjective experience of “being there”, which is linked to psychological and behavioral responses (Kaimara et al., 2022). Performance in VE is positively correlated with the level of presence induced by the environment, which is affected by the individual’s degree of immersion. Therefore, there is a relationship between immersion and presence; immersion influences presence, which in turn affects individual performance (Maneuvrier et al., 2020). In everyday life, individuals typically perceive and process information through various sensory modalities. As a result, the advancement of multisensory information including encompassing visual, auditory, tactile, olfactory, and gustatory inputs, has attracted considerable attention (Wagener et al., 2022). The realism of virtual reality can be enhanced through the provision of multisensory information. Research indicates that multisensory inputs, including auditory, tactile, gustatory, and somatic sensations, can enhance the user experience in VR (Martin et al., 2021). Furthermore, increasing any single sensory input within a VE can also enhance users’ sense of immersion.

However, studies examining the influence of multisensory information on VR experiences have predominantly focused on auditory and tactile modalities, largely due to technological feasibility and user acceptance (Shanmughapriya et al., 2024). In VR experiences, audiovisual elements serve as essential components for creating immersion. However, the introduction of haptic feedback significantly enhances emotional induction with technological advancements, thereby enhancing users’ sense of immersion and presence (Vrontos et al., 2024). Haptic feedback simulates tactile sensations from the real world, allowing users to see, hear, and “feel” the texture, temperature, and shape of objects while interacting with the virtual environment. Research demonstrates that haptic feedback from the footsteps of virtual characters in a virtual social environment enhances participants’ sense of social presence compared to conditions that rely solely on visual or audiovisual stimuli (Lee et al., 2017). This multisensory experience enhances engagement in the virtual environment, leading to increased emotional resonance. The haptic feedback activates brain regions associated with emotion, thereby enhancing users’ emotional responses. For example, when users interact with a virtual object in VR, haptic feedback creates a sensation of physical contact and strengthens emotional connections to that object. This phenomenon has shown beneficial effects in various fields, including gaming, education, and psychotherapy (Dey et al., 2018; Kono et al., 2018; Kruijff et al., 2015; Kruijff et al., 2017). VR technology that simulates height scenarios in virtual caves has been shown to effectively induce acrophobia and avoidance behaviors (Gromer et al., 2018). Additionally, the integration of wind simulation into the VE enhances immersion by stimulating skin sensations, thereby significantly enhancing participants’ fear responses (Gromer et al., 2019). Multisensory cues typically evoke more stronger physiological responses. For example, participants who received additional passive haptic feedback in a VE exhibited higher heart rates and skin conductance than those exposed only to visual stimuli (Meehan et al., 2002). Passive haptic elements within a virtual threat environment increase users’ physiological arousal, resulting in an amplified stress response. Therefore, the integration of haptic interactions in VR has the potential to enhance immersion and affect the intensity of emotional induction.

Recently, we employed a VR situational induction method to develop a fear-inducing scenario centered around high-rise buildings using graded haptic feedback (platform shaking) to manipulate threat intensity (Cong et al., 2024). In this context, we developed a physical platform that visually corresponds a transparent bridge extending 4.8 meters from the rooftop in the virtual environment. The platform’s mechanical sloshing induced by participant motion was computationally synchronized with the VR bridge’s visual dynamics (appearance, volumetric deformation, and kinematic patterns), maintaining cross-modal consistency between physical/virtual environments. Crucially, haptic feedback enhances threat realism by resolving visuo-vestibular conflicts (Gromer et al., 2019), which amplifies fear responses and their behavioral correlates. The design of our emotional induction platform explicitly prioritizes task relevance. When haptic feedback is directly consistent with environmental threats, it may enhance participants’ perception and cognitive experience of fear. Because emotional mimicry depends not only on emotional processing as such but also on how participants prioritize information (Mauersberger et al., 2025). Therefore, task-relevant sensory input more effectively modulates emotional and behavioral responses. Thus, we conceptualized haptic feedback not only as a peripheral stimulus but as a central amplifier of threat relevance. This enhanced ecological validity by integrating sensory input with the task demands of hazardous environment. This study examines how varying levels of haptic feedback (mediated through somatosensory interaction) affect fear induction in VR. The VE (e.g., height) served as the primary threat stimulus, while haptic feedback (e.g., shaking intensity) acted as a task-relevant modulator to amplify threat perception.

## 2 Materials and methods

### 2.1 Participants

Potential participants for the study were recruited through public advertisements at the university and screened for fear of heights using self-reported scores on a 10-point Likert scale. The study included 20 male volunteers (age: *M* = 22.7, SD = 3.92) with estimated fear scores between 6 and 9, targeting a population that is fearful of heights but non-clinical. Exclusion criteria included individuals with vestibular and balance impairments, as well as those with neurological or orthopedic conditions that could interfere with postural control. Additionally, participants were required to have prior experience with virtual reality environments but should not have been exposed to virtual height scenarios. Before participation, all individuals provided written informed consent and received 600 RMB as compensation upon completion of the experiment. This study received ethical approval from the Ethical Review Committee of Air Force Medical University. As an exploratory investigation, this study was not pre-registered in a public repository. All experimental materials and analytical code will be made accessible upon reasonable request. No serious adverse events were documented throughout the study duration.

### 2.2 Apparatus

The VE was developed using the Unity3D Engine, showcasing a high-rise building that reaches a height of 250 meters. This immersive experience was presented through an HTC Vive headset (HTC, Taiwan, China), which offers a resolution of 1,080 by 1,200 pixels per eye, a refresh rate of 90 Hz, and a wide field of view of 100 degrees. The experiment was performed on a computer operating on the Windows 10 64-bit system. The hardware specifications of the computer included an Intel Core i9-11900F processor, 64 GB of RAM, and an Nvidia RTX 3080 graphics card. Physiological signals were recorded using an electroencephalography (EEG) amplifier (Brain Products, Munich, Germany) and a Bluetooth physiological monitor which measured heart rate (HR), respiration rate (RESP), peripheral capillary oxygen saturation (SpO_2_), pulse rate (PR), and skin temperature (TEMP) (Tianjin Puray Instruments Ltd., Tianjin, China). The haptic physical interaction platform with a size of 4.8 m × 0.5 m was used to realize the somatosensory interaction of VR in virtual height stimulation scenes. The base of platform is equipped with two cylinder spring shock absorbers, providing vertical and lateral freedom of movement for the plate (vertical displacement ±15 cm, lateral tilt ± 8°). Motion amplitude and frequency were passively driven by participants’ actions rather than programmed actuators. This design mimics real-world unstable surfaces (e.g., swaying bridges) where haptic feedback inherently depends on user interaction.

### 2.3 Experimental design and procedures

**Figure 1:**
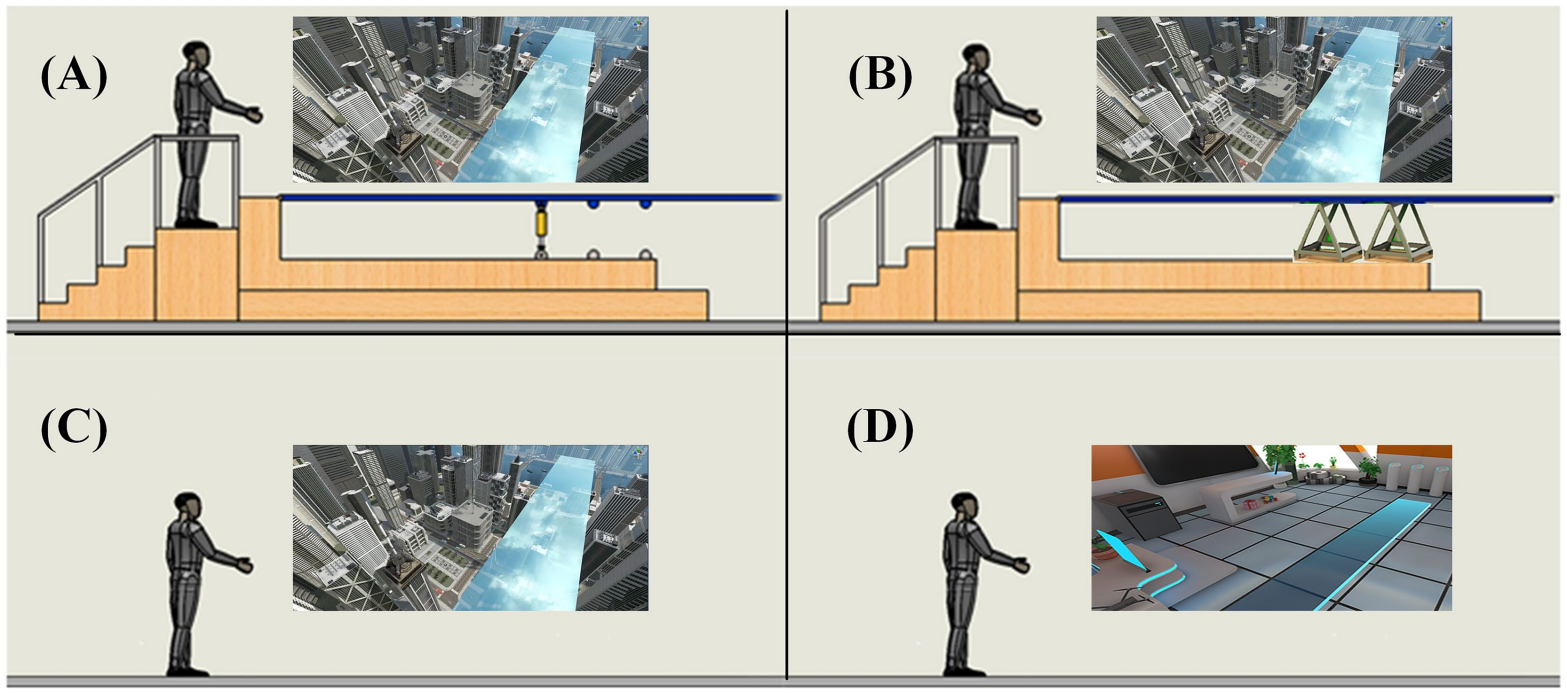
Schematic representation of the experimental setup and the four induced conditions for fear of heights. **(A)** The shaking condition was designed to instability when stepping onto a platform which equipped with a shock absorber and can give the panel freedom to move up or down. **(B)** In the stationary condition, the bottom of the platform is supported by a steel frame to ensure that it does not shake. **(C)** The ground condition refers to walking on the floor, whereas the scene maintains the VR height exposure. **(D)** The neutrality condition is a safe and quiet space where participants can walk on the floor without the fear of height exposure.

A within-subject design was employed in this experiment, with the independent variable being the level of haptic feedback in a fear of heights. Each participant was required to complete four induced conditions: neutrality, ground, stationary, and shaking, with a one-week interval between each session. The physical platform was engineered to accommodate both dynamic (shaking) and static (stationary) operational modes. Individuals experience stress and instability in the shaking condition when stepping onto a physical platform (featuring a shock absorber) paired with a virtual transparent bridge extending from a rooftop at 250 meters height, where the bridge also vibrates synchronously in the virtual environment (Figure 1A). In the stationary, no significant shaking occurred in platform after the human body stepped, while the VR environment retained identical height exposure (Figure 1B). For the ground condition, participants walked on the actual floor (no physical platform), yet the VR scene maintained the same high-altitude threat ensuring visual continuity with the shaking and stationary conditions (Figure 1C). There is still a difference in somatosensory interaction between walking on the ground and walking on the stationary platform. The neutrality condition is a safe and quiet room that acts as a non-VR-height baseline where participants experienced a non-threatening VR environment (a flat, static space without height exposure) while walking on the floor (Figure 1D). This aimed to isolate fear of heights specific responses by contrasting it with the three experimental conditions that explicitly incorporated height exposure. The contrasts between the three experimental conditions (ground, stationary, and shaking) and the neutrality condition (non-height VR baseline) are critical for isolating the specific effects of height exposure on fear induction. By comparing these conditions to a neutral, non-threatening VR environment, we can disentangle fear responses attributable to height exposure itself from baseline physiological or psychological arousal caused by VR immersion. The key characteristics and design of experimental conditions are shown in Table 1.

**Table 1:** The key characteristics and design of experimental conditions.

Condition	Walking	VR height exposure	Physical platform instability	Primary purpose
neutrality	floor	no	no	Non-VR-height baseline; controls for general VR immersion effects.
ground	floor	yes	no	VR height exposure baseline; isolates fear from visual-proprioceptive mismatch.
stationary	platform	yes	no	Tests fear induced by visual height cues + stable physical platform.
shaking	platform	yes	yes	Tests fear amplification via multisensory conflict (visual + haptic instability).

The actual experiment consisted of the fixed exposure to the four conditions which was in a crossover balanced design to eliminate the effects of experimental order. The order of the four conditions (neutrality, ground, stationary, shaking) was fully randomized for each participant using a computer-generated randomization algorithm. A balanced Latin square design ensured each experimental condition occurred once per session. This ensured balanced permutation sequences. Supplementary Table S1 presents the experimental randomization matrix design. Conditions were administered with a 1-week washout period between sessions to further mitigate potential order effects. After the arrival in the laboratory, participants read and signed the informed consent. Participants were then equipped with electrodes for ECG and EEG measurement. Before being exposed to the visual height, participants were required to fill in Positive and Negative Affect Schedule (PANAS) questionnaires and read an information letter, which described the concept of presence in VR, and how it would be rated during the experiment. Subsequently, participants were placed in the center of the VR tracking area and helped to put on the VR headsets. Following this, they were equipped with virtual reality (VR) headsets and exposed to fear-inducing stimuli. They were instructed to walk back and forth on the platform within a five-minute timeframe, all the while completing the nine-light task (Yang et al., 2023). The task was displayed upper-middle position in the subject’s VR visual field, allowing clear visibility without head movement. The interface comprised nine position translucent lights in red, yellow and green colors. This configuration mainly assessed executive functioning and sustained attentional processing. The participants were required to press the corresponding button on the VR controller when a light was lit in every 5 s. The light would turn off when the correct button was pressed or after 3 s. When completing the experiment, participants filled in another set of PANAS.

### 2.4 Psychological, physiological and behavioral measures

The PANAS is a 20-item scale designed to assess two distinct dimensions of mood: positive affect and negative affect (Watson et al., 1988). The scale includes 10 items measuring positive affect and another 10 items measuring negative affect. Respondents indicate the intensity of their feelings for each mood state using a 5-point Likert scale, with options ranging from 1 (very slightly or not at all) to 5 (extremely). The positive affect scale has demonstrated reliability, with Cronbach’ s alpha coefficients ranging from 0.86 to 0.90, indicating high internal consistency. Similarly, the negative affect scale shows reliable results, with coefficients ranging from 0.84 to 0.87 (Watson et al., 1988). Some study isolate fear-related items from PANAS to assess context-specific emotional responses (Eadeh et al., 2020; Ortuño-Sierra et al., 2015). In this experiment, the degree of fear was assessed by summing the scores of three negative items: scared, nervous, and afraid.

EEG data is collected using a 32-channel amplifier with the impedance of each electrode maintained below 10 kΩ, continuously sampled at a frequency of 500 Hz. The electrodes are positioned according to the international 10/20 system. Additional physiological signals that can be collected by the Bluetooth physiological monitoring device. HR and RESP was measured using an electrocardiogram obtained from three Ag/AgCl electrodes positioned under the right collarbone, on the lower left costal arch, and on the lower left back. The PR is obtained by the sensor of SpO_2_ on the finger. The TEMP sensor was positioned at the center of the thorax.

During the experiment, participants’ locations within the VE were monitored in real-time. Their movement distance (MD) on the physical platform was recorded using two HTC VIVE trackers attached to the insteps of both feet. Participants were instructed to press the designated button on the VR controller whenever a light illuminated, with a new light appearing every 5 s. The light extinguished either when the correct button was pressed or after 3 s. Throughout the experiment, participants’ reaction times (RT) and accuracy rates (ACC) were automatically recorded for analysis. Eye-tracking data were collected using the HTC Vive Pro Eye, which employs a 5-point calibration method for accurate individual gaze tracking. The output data included timestamps, saccade amplitude (calculated from the gaze origin), eye openness, and pupil diameter, among other metrics (Schuetz and Fiehler, 2022).

### 2.5 EEG preprocessing and feature extraction

A 32-Channel conventional gel injection electrode cap (Brain Products, Munich, Germany) was used to ensure stability during the experimental procedures. Digital filters were applied to remove unwanted frequencies from the EEG signals. High-pass filters eliminate low-frequency artifacts, such as DC drift and slow physiological changes, while low-pass filters reduce high-frequency noise, including muscle artifacts and line interference. A Butterworth bandpass filter ranging from 0.5 Hz to 49 Hz is applied to preserve potential high-gamma (>30 Hz) components linked to cognitive processing for further analysis. 50 Hz trap filtering is also applied to further remove powerline interference. Eye movements, especially blinking and saccades, frequently contaminate EEG recordings. Independent Component Analysis (ICA) was used to identify and remove these artifacts. ICA decomposes the EEG signal into independent components, facilitating visual inspection and removal of those associated with ocular activity. Muscular artifacts were addressed through a combination of automated artifact detection algorithms and manual inspection. Components with high-frequency bursts or significant temporal variability, indicative of muscular activity, were excluded. On average, 2 to 5 independent components are eliminated for each participant (Pei et al., 2025). Continuous EEG data were referenced to an average and segmented into 2-s epochs (typically lasting from 1 to 4 s) for subsequent analysis. During the VR experiment, high-amplitude noise was observed in electrodes positioned over the temporo-parietal, occipital, and frontal regions. This artifact was primarily attributed to participant body movements causing neck and jaw muscle activation, and mechanical pressure exerted by the VR headset. Therefore, several brain electrodes (Fp1, FT9, TP9, O1, Oz, O2, TP10, FT10, Fp2) were removed due to poor signal quality during the collection of brain electrical activity. Consequently, 25 electrodes (i.e., Fz, F3, F7, FC5, FC1, C3, T7, CP5, CP1, Pz, P3, P7, P4, P8, CP6, Cz, C4, T8, FC6, F4, and F8) were retained for further data analysis.

Band power is a widely used frequency domain feature since it is a well-established method (Jenke et al., 2014). Various algorithms have been proposed to calculate band power, including the fast Fourier transform (FFT), short-time Fourier transform (STFT), and Welch’ s method for estimating spectral power density. In this study, power spectral densities (PSD) were extracted from 2-s epochs using Welch’s estimator. Sample entropy (SE) quantifies the regularity of a time series by calculating the negative logarithm of the conditional probability of two sequences. SE has become an essential tool for quantifying the regularity and complexity of dynamical systems in various fields, including neuroscience and physiology (Patidar, 2024). Due to their robustness over time and low computational cost, power spectrum and sample entropy were chosen as EEG features and categorized into four frequency bands: delta (*δ*, 1–3 Hz), theta (*θ*, 4–7 Hz), alpha (*α*, 8–12 Hz), and beta (*β*, 13–30 Hz) for further analysis.

### 2.6 Data analysis

The SPSS Statistics 23.0 software (IBM, Armonk, NY, USA) was applied in this study. Statistical methods, including repeated measures analysis of variance (RMANOVA) and paired t-tests, were employed when the collected data demonstrated normal distribution and homogeneity of variance. The obtained data were adjusted for multiple comparisons using the false discovery rate (FDR). Before conducting RMANOVA, we tested the assumptions of sphericity using Mauchly’ s test. In cases where the assumption of sphericity was violated (*p <* 0.05), Greenhouse–Geisser corrections were applied to adjust the degrees of freedom accordingly. Statistical significance was set at *p <* 0.05 for all tests.

## 3 Results

### 3.1 Subjective fear perception

**Figure 2:**
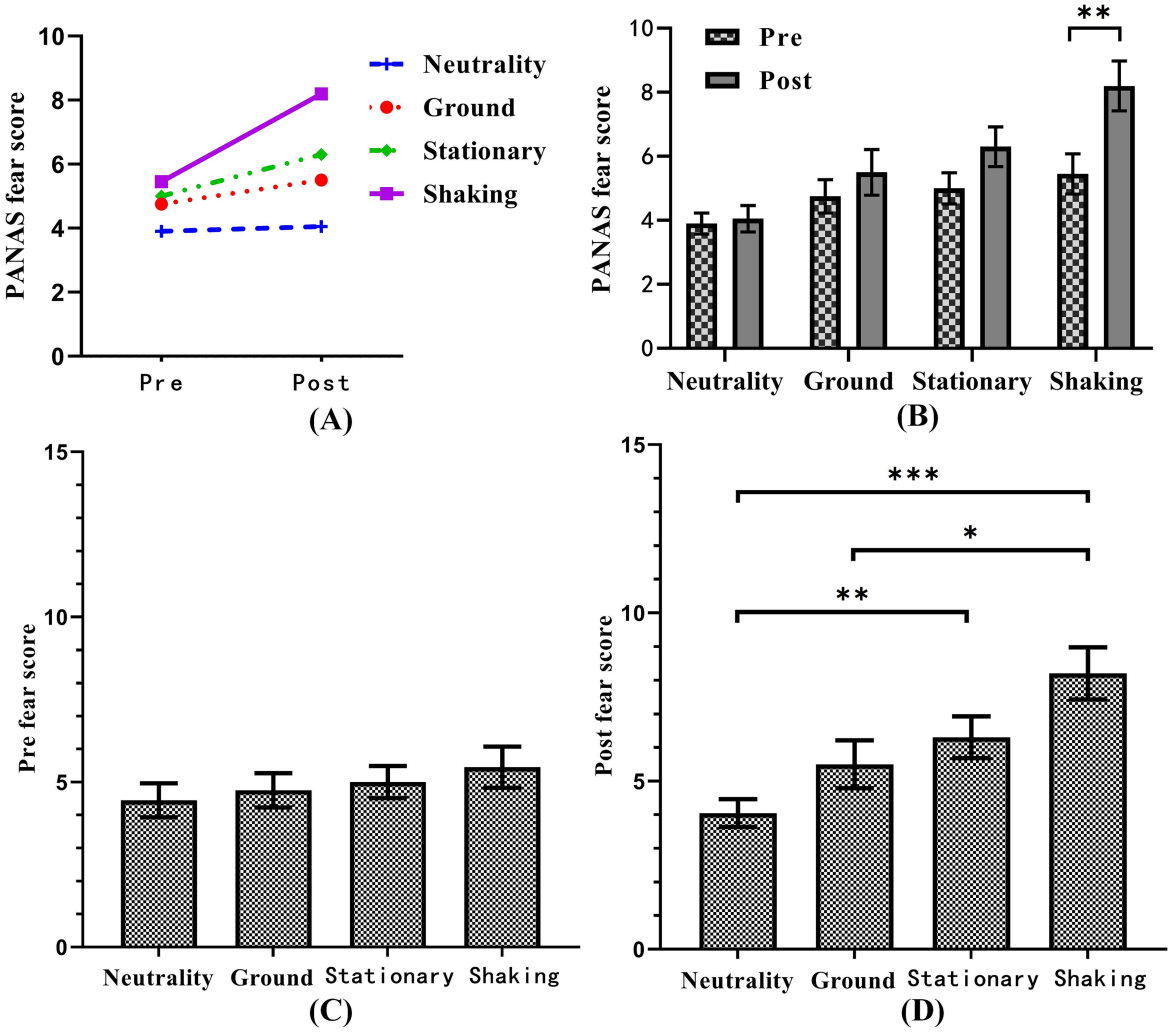
The scores of psychological measures in the four induced conditions. **(A)** The interaction effect analysis between the measurement stage and the induced condition. **(B)** The simple effects analysis of the measurement stage. **(C)** The pre-test fear score from the four induced condition. **(D)** The post-test fear score from the four induced condition. *p < 0.05, **p < 0.01, ***p < 0.001.

The subjective scale was collected both before and after the experiment, allowing for the application of a Two-Way RMANOVA to assess the effects of measurement stages and induced conditions. The PANAS fear score exhibited a significant difference (*F* = 3.683, *p <* 0.05). Therefore, an interaction effect exists between the measurement stage and the induced condition, as illustrated in Figure 2A. To further elucidate this interaction, we performed simple main effects analyses for each factor level. The simple effects analysis of the measurement stage revealed no significant differences in the PANAS fear scores in the neutrality condition (*T* = 0.818, *p >* 0.05), ground condition (*T* = −1.009, *p >* 0.05), and stationary condition (*T* = −1.782, *p >* 0.05) (Figure 2B). In the shaking condition, a significant difference was observed between pre-measurement and post-measurement (*T* = −2.866, *p <* 0.01). This suggests that the effect of the measurement stage on the PANAS fear score was contingent upon the induced condition. Another simple effects analysis of the induced conditions, as shown in Figures 2C,D, revealed no significant difference in the PANAS fear scores at pre-measurement (*F* = 2.193, *p >* 0.05). However, a significant difference was observed at post-measurement (*F* = 13.046, *p <* 0.001). These findings indicate that the effect of the induced conditions on the PANAS fear score varied across different measurement stages.

### 3.2 Peripheral physiological arousal

The One-Way RMANOVA indicated significant differences in heart rate across various induced conditions (*p <* 0.05, *F* = 2.891, *η*^2^ = 229.45), as presented in Table 2, indicating that the intensity of shaking significantly affects heart rate. Specifically, Post-hoc analyses using the Bonferroni method showed significant differences between the shaking condition and the neutrality, ground, and stationary conditions (*p* < 0.05 for all). There was no significant difference in other pairwise comparisons (*p >* 0.05). Additionally, the statistical analysis showed no significant differences in PR, RESP, SpO2, and TEMP (*p* > 0.05), suggesting that fear-induced conditions did not significantly impact these indices.

**Table 2:** Data statistics of the peripheral physiological in the four induced conditions.

Parameter	Induced conditions	M	SD	SS	df	MS	F	*p*	*η* ^2^
HR	neutrality	83.546	9.351	688.351	3.000	229.450	2.891*	0.044	0.138
	ground	82.263	10.570						
	stationary	83.055	13.019						
	shaking	89.824	10.031						
PR	neutrality	85.436	9.765	288.082	3.000	96.027	1.686	0.181	0.086
	ground	84.437	10.222						
	stationary	85.859	12.177						
	shaking	89.579	10.755						
RESP	neutrality	24.067	3.834	39.019	2.101	18.576	1.358	0.270	0.070
	ground	24.680	3.620						
	stationary	24.627	3.930						
	shaking	26.017	4.586						
SpO2	neutrality	97.192	1.102	7.390	1.333	5.546	0.550	0.514	0.030
	ground	97.001	3.825						
	stationary	97.331	0.807						
	shaking	97.842	1.173						
TEMP	neutrality	33.726	0.937	0.431	3.000	0.144	0.186	0.906	0.010
	ground	33.637	0.983						
	stationary	33.517	1.243						
	shaking	33.594	1.313						

Heart rate (HR), respiration rate (RESP), peripheral capillary oxygen saturation (SpO2), pulse rate (PR), skin temperature (TEMP). The significance levels are marked as follows: *p < 0.05.

### 3.3 Brain activity dynamics

#### 3.3.1 Power spectrum analysis

**Figure 3:**
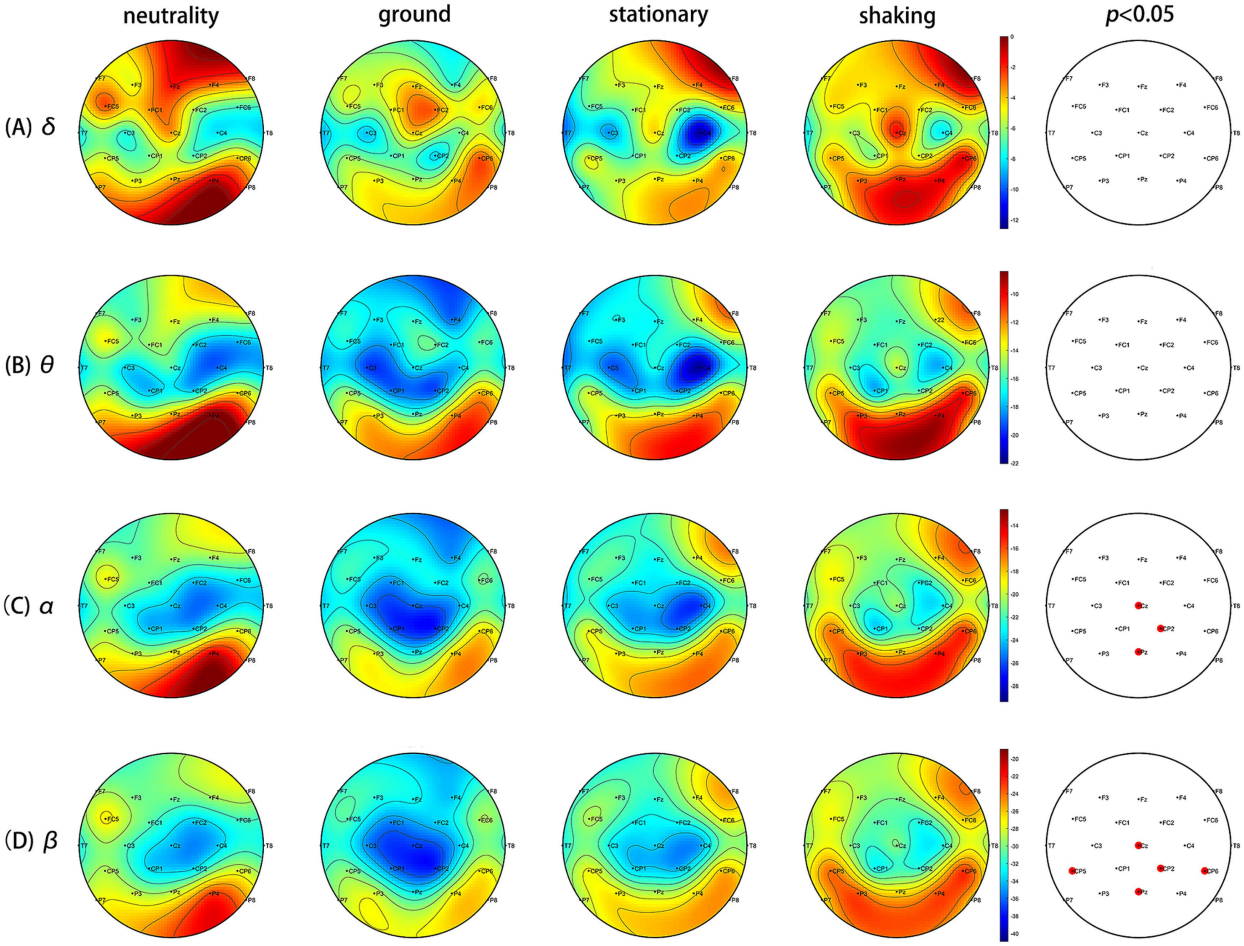
The brain topographic maps based on the power spectral density (PSD) index of the four induced conditions. **(A)** *δ* band (1–3 Hz) PSD differences (black dots: FDR correction *p* > 0.05). **(B)** *θ* band (4–7 Hz) PSD differences (black dots: FDR correction *p* > 0.05). **(C)** *α* band (8–12 Hz) PSD differences. There were significant differences at Cz, CP2, and Pz after FDR correction (red dots: *p* < 0.05). **(D)** *β* band (13–30 Hz) PSD differences. There were significant differences at Cz, CP2, Pz, CP5 and CP6 after FDR correction (red dots: *p* < 0.05).

**Figure 4:**
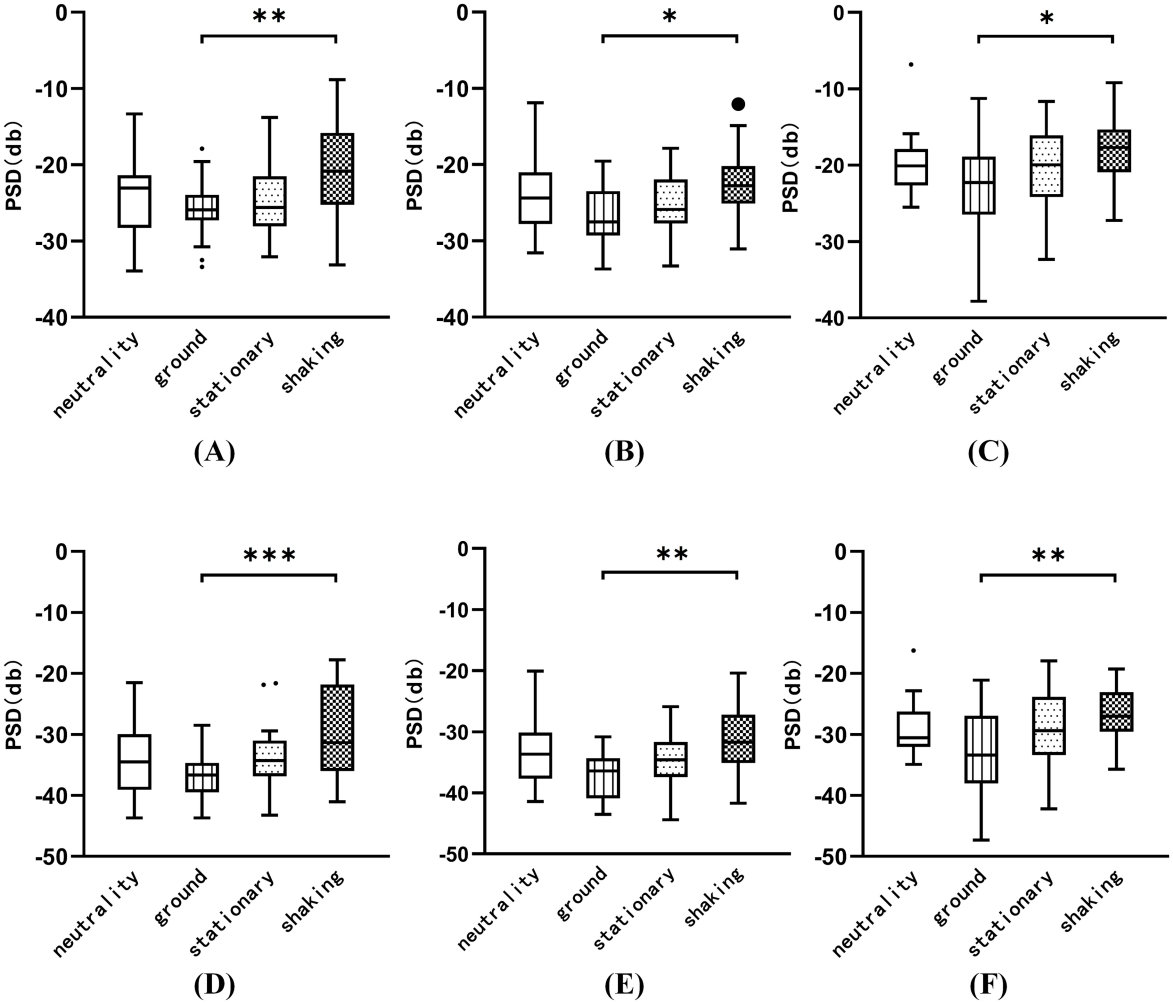
The PSD of the Cz, CP2, and Pz channels was analyzed across the four induced conditions in the α and β frequency bands. **(A)** The PSD of the Cz channels in the α band. **(B)** The PSD of the CP2 channels in the α band. **(C)** The PSD of the Pz channels in the α band. **(D)** The PSD of the Cz channels in the β band. **(E)** The PSD of the CP2 channels in the β band. **(F)** The PSD of the Pz channels in the β band. *p < 0.05, **p < 0.01, ***p < 0.001.

The brain topographic maps based on the PSD index of the four induced conditions were created, as shown in Figure 3. The results showed significant differences in EEG channels across the four induced conditions, mainly in the Cz, CP2, and Pz channels in the *α* band, and the CP5, CP6, Cz, CP2, and Pz channels in the *β* band (FDR correction, *p* < 0.05). Figures 4A–C show that the PSD of the Cz, CP2, and Pz channels in the shaking is significantly higher than that in the ground condition (*p* < 0.05) in the α band. Figures 4D–F indicate that the PSD of the Cz, CP2, and Pz channels in the shaking condition also exhibits significantly higher values in the β band compared to the ground condition (*p* < 0.05).

#### 3.3.2 Sample entropy analysis

**Figure 5:**
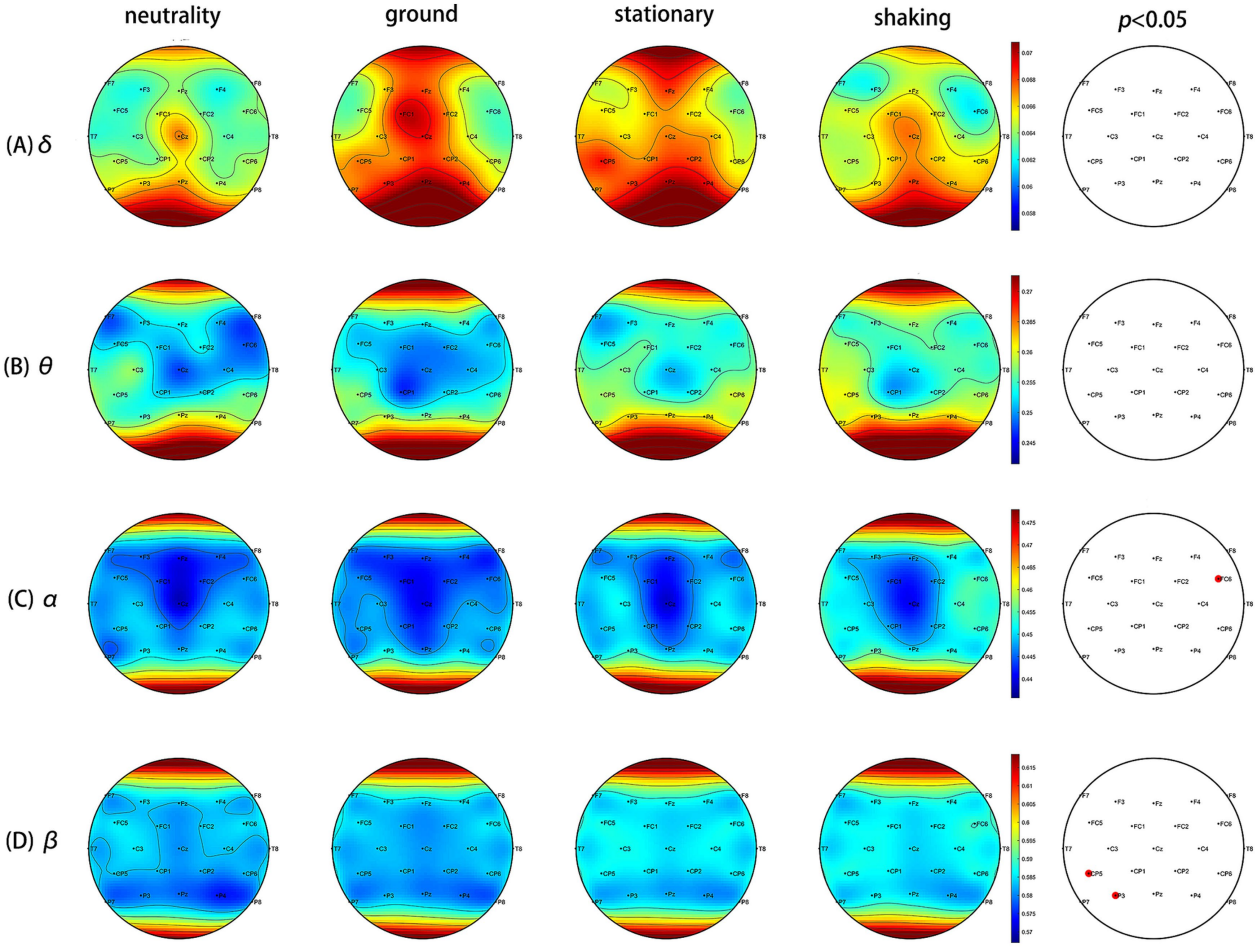
The brain topographic maps based on sample entropy (SE) index of the four induced conditions. **(A)** δ band (1–3 Hz) SE differences (black dots: FDR correction *p* > 0.05). **(B)** θ band (4–7 Hz) SE differences (black dots: FDR correction *p* > 0.05). **(C)** α band (8–12 Hz) SE differences. There were significant differences at FC6 after FDR correction (red dots: *p* < 0.05). **(D)** β band (13–30 Hz) SE differences. There were significant differences at CP5 and P3 after FDR correction (red dots: *p* < 0.05).

**Figure 6:**
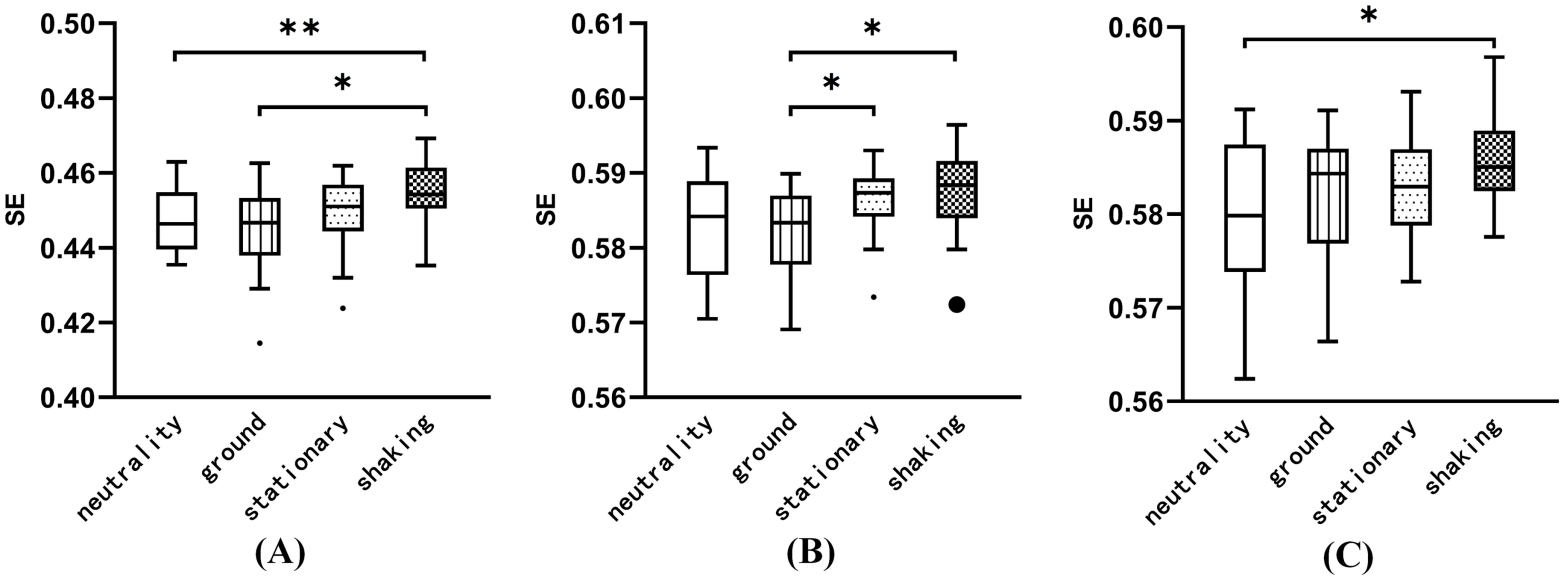
The SE of the FC6, CP5, and P3 channels was analyzed across four induced conditions in the α and β frequency bands. **(A)** The SE of the FC6 channels in the α band. **(B)** The SE of the CP5 channels in the β band. **(C)** The SE of the P3 channels in the β band. *p < 0.05, **p < 0.01.

The brain topographic maps based on SE index of the four induced conditions were drawn in Figure 5. The results revealed significant differences in EEG channels across the four induced conditions, particularly in the FC6 channel in the α band and the CP5 and P3 channels in the β band (FDR correction, *p* < 0.05). Figure 6A shows that the SE in the FC6 channel in the shaking is significantly higher than that in the neutrality and ground conditions (*p* < 0.05) in the α band. Figure 6B indicates that the SE in the CP5 channel in the shaking is significantly elevated compared to the ground condition in the β band (*p* < 0.05). Additionally, the SE in the stationary condition is significantly greater than that in the ground condition in the β band (*p* < 0.05). In the P3 channel, the SE in the shaking is significantly elevated compared to the neutrality condition in the β band (*p* < 0.05), as shown in Figure 6C.

### 3.4 Behavioral shifts

#### 3.4.1 Eye movement analysis

Table 3 presents the eye movement parameters across the four induced conditions. In the shaking condition, participants demonstrated a significantly larger pupil diameter in the left eye compared to the neutral, ground, and stationary conditions (*F* = 5.942, *p* < 0.01). Moreover, the pupil diameter of the right eye in the shaking condition was significantly larger than in the neutral and stationary conditions (*F* = 3.148, *p* < 0.05). As the induced conditions changed, the trend in pupil diameter for both eyes remained consistent. There were no significant differences in saccade amplitude and eye openness indices (*p >* 0.05).

**Table 3:** Data statistics of the eye movement in the four induced conditions.

Parameter	Induced conditions	M	SD	SS	df	MS	F	*p*	*η* ^2^
Left eye saccade amplitude	neutrality	48.178	4.584	86.934	1.939	44.835	2.939	0.067	0.134
	ground	47.876	4.927						
	stationary	50.413	5.977						
	shaking	49.645	6.539						
Right eye saccade amplitude	neutrality	46.511	6.302	21.981	3.000	7.327	0.588	0.625	0.030
	ground	46.510	4.390						
	stationary	47.530	5.180						
	shaking	46.114	4.563						
Left eye pupil diameter	neutrality	3.805	0.798	3.281	3.000	1.094	5.942**	0.001	0.238
	ground	3.873	0.827						
	stationary	4.027	1.027						
	shaking	4.331	1.020						
Right eye pupil diameter	neutrality	3.788	0.838	2.198	2.322	0.947	3.148*	0.046	0.142
	ground	3.852	0.820						
	stationary	3.862	1.215						
	shaking	4.211	1.162						
Left eye openness	neutrality	0.870	0.073	0.012	2.125	0.006	0.928	0.408	0.047
	ground	0.864	0.096						
	stationary	0.896	0.061						
	shaking	0.883	0.064						
Right eye openness	neutrality	0.855	0.019	0.021	3.000	0.007	2.048	0.117	0.097
	ground	0.888	0.009						
	stationary	0.873	0.018						
	shaking	0.898	0.013						

The significance levels are marked as follows: *p < 0.05, **p < 0.01.

#### 3.4.2 Cognitive and movement performance

**Figure 7:**
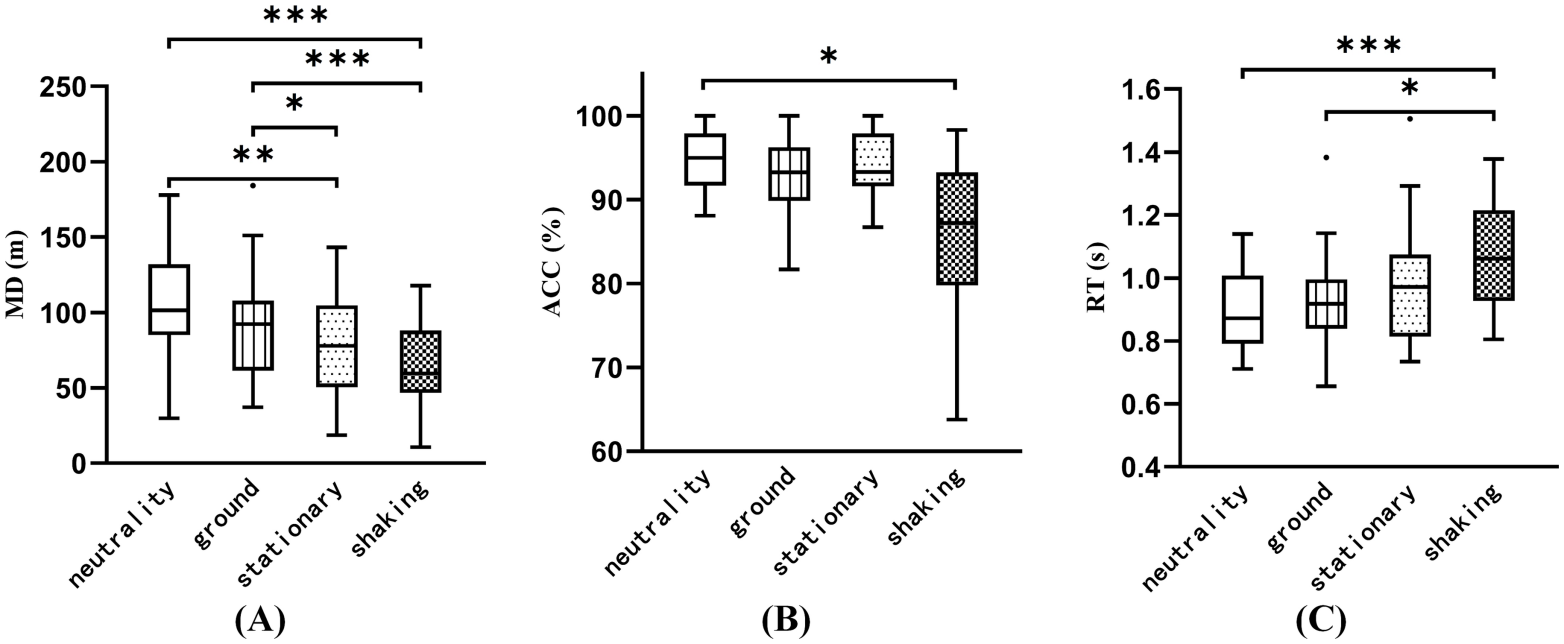
The performance of behavioral and cognitive parameters in the four induced conditions. **(A)** The movement distance calculated from the participants’ location while walking on the platform. **(B,C)** The Nine-light task performance (ACC and RT). *p < 0.05, **p < 0.05, ***p < 0.05.

Figure 7A shows a significant difference in MD (*F* = 15.782, *p* < 0.001), where the shaking condition exhibits a lower value than both the neutrality and ground conditions (*p* < 0.001). Additionally, the MD in the stationary condition is significantly lower than in the neutrality condition (*p* < 0.001). Figure 7B indicates a significant difference in ACC (*F* = 5.707, *p* < 0.01), with the shaking condition showing a lower value than the neutrality condition (*p* < 0.05). Figure 7C indicates a significant difference in RT (*F* = 8.230, *p* < 0.001), with the shaking condition showing a higher value than the neutrality and ground conditions (*p* < 0.05).

## 4 Discussion

This study investigates how affective stimuli, amplified via graded haptic feedback (platform shaking), reconfigure motor behavior by modulating attentional resources and action pathways. The subjective assessments using the PANAS revealed a significant difference in fear scores specifically for the shaking condition compared to other conditions, highlighting its distinct impact. This suggests that the haptic feedback provided in the shaking condition effectively heightened the participants’ sense of fear. This aligns with existing literature indicating that incorporating haptics into VE can increase sensory fidelity and provide powerful and immersive experiences (Venkatesan et al., 2023). The study on the effects of haptics in spider virtual reality exposure therapy (VRET) revealed that the haptic condition was more effective in inducing anxiety compared to both the control group and a similar scenario without haptics (Brice et al., 2021). A study of VR games found that haptic feedback made them feel more authentic about the action and threats in the game (Venkatesan et al., 2023). Therefore, haptic feedback simulates physical sensations, enhancing the users’ sense of presence and emotional connection in the virtual world. The absence of significant differences in neutrality, ground, and stationary conditions emphasize the specific role of haptics in modulating fear perceptions.

Physiologically, the heart rate data further supported the subjective findings, with significant elevations observed in the shaking condition compared to others. This physiological arousal aligns with the heightened fear scores and suggests an autonomic nervous system response to perceived threats in VR (Bzdúšková et al., 2022). When simulating a high-altitude scenario in VR, participants showed significant physiological fear responses, including changes in heart rate and skin conductance (Diemer et al., 2016). In addition, the results of another study also showed that high exposure in VR significantly increased participants’ subjective fear levels, and this was confirmed by heart rate variability (HRV) analysis (Kristína et al., 2024). Notably, other physiological indices such as pulse rate, respiration, and skin temperature did not exhibit significant changes, indicating that heart rate may be a more sensitive marker of acute fear responses in VR environments.

The EEG data provided additional insights into the neural correlates of fear induction. The increased power spectral density (PSD) values in the *α* and *β* in the shaking condition, particularly in overlapping channels such as Cz, CP2, and Pz, suggest heightened cortical activity associated with processing fear-related stimuli. Heightened β activity suggests hypervigilance mediated by the amygdala-prefrontal circuitry (Davis and Whalen, 2001; Pessoa, 2008). Increased *β* synchronization likely represents amplified sensory processing in cortical threat detection networks, particularly the salience network anchored in the insula and dorsal anterior cingulate (Seeley et al., 2007). This aligns with our observation of β increases at CP2/Pz which involved in multisensory integration of threat cues (Mobbs et al., 2010). Paradoxically, elevated α activity co-occurred with heightened β. The α is generally associated with relaxation, wakefulness, and brain inhibition, while the β is highly associated with alertness, arousal of emotion (Li and Zheng, 2021). One research indicates a significant correlation between the severity of acrophobia and the mean α and β activity and this suggests an increase in alertness and cognitive engagement when exposed to fear-inducing stimuli (Russo et al., 2024). One evidence suggests that increased α activity is associated with reduced attentional responses to specific stimuli, such as spiders, indicating that α may play an inhibitory role in unconscious defense mechanisms (Bazan, 2017). While participants reported consciously redirecting attention to the nine-light task to manage fear, their reduced task accuracy suggests a trade-off between fear regulation and cognitive performance. The dual α and β enhancement reveals a neural efficiency trade-off. Participants’ focus on the task was effortful and resource-limited. Prefrontal resources allocated to fear regulation (indexed by frontoparietal β activity) likely compromised executive function required for the nine-light task. This interpretation aligns with Pessoa’s “network competition model”: amygdala-driven arousal consumes prefrontal resources normally supporting cognitive control (Pessoa, 2008). The significant accuracy decline during shaking condition exemplifies this resource reallocation.

Evidence indicates that regions near the central parietal lobe, including electrodes Cz, CP1, CP2, and Pz, exhibit the strongest efferent information flow during danger recognition, suggesting their involvement in identifying and processing potentially threatening stimuli (Wei et al., 2024). Although the study focused on danger recognition, it can be speculated that these brain regions may also play an important role in fear responses, as fear is often associated with the perception and response to potential threats. Research has shown that emotional intensity and personality type significantly impact the SE as emotional intensity increases (Wan et al., 2023). The CP5 is the common channel of the two EEG indices of PSD and SE in β band. A study on music emotion recognition utilizing EEG entropy features found that, based on statistical analysis of the optimal channel number from 30 subjects, the principal component channel in the central brain region is CP5, which is closely associated with emotional responses (Xie et al., 2022). Elevated SE values in CP5 further imply increased complexity and engagement of neural circuits in response to the shaking condition. Haptic feedback may enhance the effect of VR fear induction in the shaking condition, as increased tactile cues can enrich the sense of presence (Gromer et al., 2019). Compared to the neutral condition, both the ground and stationary conditions can also induce fear. However, the low amplitude of shaking results in weak feedback, which leads to a minimal evoked effect.

Significant differences were also observed in pupil diameter, MD, RT, and ACC across the four induced conditions. Inducing fear in the shaking condition significantly affected participants’ cognition and behavior, leading to increased avoidance behavior and decreased performance. The enlargement of pupil diameter indicates heightened arousal and attentional demands. The increase in pupil size associated with fear results from the activation of the sympathetic nervous system, which causes the dilator pupillae muscle to contract, leading to pupil dilation (Mathôt, 2018). Compared to positive emotional stimulation, individuals exhibit greater sensitivity to negative emotional stimulation, resulting in increased pupil dilation that persists for a longer duration (Derksen et al., 2018; Oliva and Anikin, 2018). The lower ACC along with increased RT, indicate a significant disruption in nine-light task performance due to the cognitive load imposed by induced fear. These findings are consistent with cognitive-emotional interference theories (Bishop, 2007; Pessoa, 2009), which propose that high-arousal negative states like fear compete for shared attentional and executive resources, diminishing capacity for goal-directed tasks. Specifically, during the shaking condition, participants exhibited a significant increase in fear levels. This heightened fear state likely activated neurobehavioral avoidance mechanisms, reducing MD as participants subconsciously avoided physical interaction with the threatening virtual environment. This avoidance behavior indicates an automatic defense response after being stimulated by fear. The increased pupil diameter which is an autonomic arousal index, further supports this subconscious defensive response. Concurrently, the fear state likely consumed cognitive resources, impairing sustained attention and working memory necessary for efficient performance in the nine-light task, thus degrading ACC and increasing RT. Therefore, these behavioral metrics collectively demonstrate that fear diminishes the cognitive resources necessary for task completion, while high-arousal stimuli typically impair task performance (Battaglia et al., 2021). Our findings demonstrate that haptic feedback as task-relevant affective stimuli significantly altered motor behavior.

Comprehensive analysis results of subjective fear perception, physiology arousal and behavioral performance suggests that the haptic feedback provided in the shaking condition effectively heightened participants’ sense of fear compared to other conditions. The haptic feedback amplifies affective threat stimuli, creating a paradigm to dissect emotion-action interactions. By enhancing ecological realism (e.g., shaking platform synchronizing with visual height cues), haptics intensifies perceived threat credibility (Brelet and Gaffary, 2022; Kim et al., 2019; Venkatesan et al., 2023), triggering dual motor consequences: conscious appraisal of threats (e.g., shaking as part of the VR environment) competes for prefrontal resources, degrading executive motor control. While subconscious threat detection potentiates reflexive motor avoidance independent of task goals. This dissociation exemplifies how affective stimuli bifurcate motor behavior: conscious appraisal impairs goal-directed actions, while subconscious threat detection primes defensive maneuvers. Future studies should explore demographic variables (e.g., gender differences in affective-motor pathways) and explore threat relevance (e.g., haptics as distractor vs. task signal) to advance the mechanism of how emotions govern actions.

## 5 Conclusion

This study establishes haptic feedback as a potent affective stimulus amplifier that directly reconfigures motor behavior through distinct neurocognitive pathways. Our integration of physiological, behavioral, and subjective measures provides a mechanistic model for affective-motor competition, demonstrating how somatic threat cues translate into altered actions. From an application standpoint, our findings provide valuable insights for the development of VR-based applications in therapy, emotional training systems, and real-world stress simulations. Future research should explore the long-term behavior effects of repeated exposure to such stimuli, as well as their specific applications across various domains. Expanding the range of physiological and behavioral measures could also better explore the relationship between emotional stimuli and motor behavior.

## Data Availability

The raw data supporting the conclusions of this article will be made available by the authors, without undue reservation.

## References

[ref1] Anglin J. M. Sugiyama T. Liew S. L. (2017). Visuomotor adaptation in head-mounted virtual reality versus conventional training. Sci. Rep. 7:45469. doi: 10.1038/srep45469, PMID: 28374808 PMC5379618

[ref2] Battaglia S. Serio G. Scarpazza C. D'Ausilio A. Borgomaneri S. (2021). Frozen in (e)motion: how reactive motor inhibition is influenced by the emotional content of stimuli in healthy and psychiatric populations. Behav. Res. Ther. 146:103963. doi: 10.1016/j.brat.2021.103963, PMID: 34530318

[ref9001] Battaglia S. Cardellicchio P. Di Fazio C. Nazzi C. Fracasso A. Borgomaneri S.. (2022). The influence of vicarious fear-learning in “Infecting” reactive action inhibition. Front. Behav. Neurosci. 16:946263. doi: 10.3389/fnbeh.2022.946263 35941933 PMC9355887

[ref3] Bazan A. (2017). Alpha synchronization as a brain model for unconscious defense: an overview of the work of Howard Shevrin and his team. Int. J. Psychoanal. 98, 1443–1473. doi: 10.1111/1745-8315.12629, PMID: 28247941

[ref4] Bell I. H. Nicholas J. Alvarez-Jimenez M. Thompson A. Valmaggia L. (2020). Virtual reality as a clinical tool in mental health research and practice. Dialogues Clin. Neurosci. 22, 169–177. doi: 10.31887/DCNS.2020.22.2/lvalmaggia, PMID: 32699517 PMC7366939

[ref5] Bishop S. J. (2007). Neurocognitive mechanisms of anxiety: an integrative account. Trends Cogn. Sci. 11, 307–316. doi: 10.1016/j.tics.2007.05.008, PMID: 17553730

[ref6] Brelet L. Gaffary Y. (2022). Stress reduction interventions: a scoping review to explore progress toward use of haptic feedback in virtual reality. Front. Virtual Real. 3:900970. doi: 10.3389/frvir.2022.900970

[ref7] Brice D. Gibson Z. McGuinness F. Rafferty K. (2021). Using ultrasonic haptics within an immersive spider exposure environment to provide a multi-sensorial experience. Front. Virtual Real. 2:707731. doi: 10.3389/frvir.2021.707731

[ref8] Browning M. H. E. M. Mimnaugh K. J. van Riper C. J. Laurent H. K. LaValle S. M. (2020). Can simulated nature support mental health? Comparing short, single-doses of 360-degree nature videos in virtual reality with the outdoors. Front. Psychol. 10:2667. doi: 10.3389/fpsyg.2019.02667, PMID: 32010003 PMC6974516

[ref9] Bzdúšková D. Marko M. Hirjaková Z. Kimijanová J. Hlavačka F. Riečanský I. (2022). The effects of virtual height exposure on postural control and psychophysiological stress are moderated by individual height intolerance. Front. Hum. Neurosci. 15:773091. doi: 10.3389/fnhum.2021.773091, PMID: 35095450 PMC8789875

[ref10] Cong L. Yu X. Huang M. Sun J. Lv H. Zhang T.. (2024). Enhancing emotion regulation: investigating the efficacy of transcutaneous electrical acupoint stimulation at PC6 in reducing fear of heights. Front. Psychol. 15:1371014. doi: 10.3389/fpsyg.2024.1371014, PMID: 38633874 PMC11021653

[ref11] Davis M. Whalen P. J. (2001). The amygdala: vigilance and emotion. Mol. Psychiatry 6, 13–34. doi: 10.1038/sj.mp.4000812, PMID: 11244481

[ref12] Derksen M. van Alphen J. Schaap S. Mathot S. Naber M. (2018). Pupil mimicry is the result of brightness perception of the Iris and pupil. J. Cogn. 1:32. doi: 10.5334/joc.34, PMID: 31517205 PMC6634366

[ref13] Dey A. Chen H. Billinghurst M. Lindeman R. W. Data from: Effects of manipulating physiological feedback in immersive virtual environments. (2018) Available online at:https://go.exlibris.link/CkzMv1p0

[ref14] Diemer J. Lohkamp N. Mühlberger A. Zwanzger P. (2016). Fear and physiological arousal during a virtual height challenge—effects in patients with acrophobia and healthy controls. J. Anxiety Disord. 37, 30–39. doi: 10.1016/j.janxdis.2015.10.007, PMID: 26600469

[ref15] Eadeh H. Breaux R. Langberg J. M. Nikolas M. A. Becker S. P. (2020). Multigroup multilevel structure of the child and parent versions of the positive and negative affect schedule (PANAS) in adolescents with and without ADHD. Psychol. Assess. 32, 374–382. doi: 10.1037/pas0000796, PMID: 31886686 PMC7093246

[ref16] Gromer D. Madeira O. Gast P. Nehfischer M. Jost M. Müller M.. (2018). Height simulation in a virtual reality CAVE system: validity of fear responses and effects of an immersion manipulation. Front. Hum. Neurosci. 12:372. doi: 10.3389/fnhum.2018.00372, PMID: 30319376 PMC6167601

[ref17] Gromer D. Reinke M. Christner I. Pauli P. (2019). Causal interactive links between presence and fear in virtual reality height exposure. Front. Psychol. 10:141. doi: 10.3389/fpsyg.2019.00141, PMID: 30761054 PMC6363698

[ref18] Hofstee G. Jansen P. G. W. De Lange A. H. Spisak B. R. Swinkels M. (2021). The cognitive costs of managing emotions: a systematic review of the impact of emotional requirements on cognitive performance. Work Stress. 35, 301–326. doi: 10.1080/02678373.2020.1832608

[ref19] Jenke R. Peer A. Buss M. (2014). Feature extraction and selection for emotion recognition from EEG. IEEE Trans. Affect. Comput. 5, 327–339. doi: 10.1109/TAFFC.2014.2339834

[ref20] Jung N. Wranke C. Hamburger K. Knauff M. (2014). How emotions affect logical reasoning: evidence from experiments with mood-manipulated participants, spider phobics, and people with exam anxiety. Front. Psychol. 5:570. doi: 10.3389/fpsyg.2014.00570, PMID: 24959160 PMC4050437

[ref21] Kaimara P. Oikonomou A. Deliyannis I. (2022). Could virtual reality applications pose real risks to children and adolescents? A systematic review of ethical issues and concerns. Virtual Real. 26, 697–735. doi: 10.1007/s10055-021-00563-w, PMID: 34366688 PMC8328811

[ref22] Kanske P. Kotz S. A. (2011). Emotion triggers executive attention: anterior cingulate cortex and amygdala responses to emotional words in a conflict task. Hum. Brain Mapp. 32, 198–208. doi: 10.1002/hbm.21012, PMID: 20715084 PMC6870409

[ref23] Kim Y. I. Jung S. Min S. Seol E. Seo S. Hur J.. (2019). Visuo-haptic-based multimodal feedback virtual reality solution to improve anxiety symptoms: a proof-of-concept study. Psychiatry Investig. 16, 167–171. doi: 10.30773/pi.2018.12.25.1, PMID: 30808124 PMC6393753

[ref24] Kono M. Miyaki T. Rekimoto J. Spencer S. N. Data from: In-pulse: Inducing fear and pain in virtual experiences. (2018) Available online at:https://go.exlibris.link/1nNfXBfh

[ref25] Kristína V. Dagmar S. Oto J. Lenka J. Kateřina B. Vojtěch J. (2024). Virtual reality exposure effect in acrophobia: psychological and physiological evidence from a single experimental session. Virtual Real. 28:137. doi: 10.1007/s10055-024-01037-5

[ref26] Kruijff E. Marquardt A. Trepkowski C. Schild J. Hinkenjann A.. Data from: Enhancing user engagement in immersive games through multisensory cues. (2015) Available online at:https://go.exlibris.link/4mbW45Gx

[ref27] Kruijff E. Marquardt A. Trepkowski C. Schild J. Hinkenjann A. (2017). Designed emotions: challenges and potential methodologies for improving multisensory cues to enhance user engagement in immersive systems. Vis. Comput. 33, 471–488. doi: 10.1007/s00371-016-1294-0

[ref28] Lee M. Bruder G. Welch G. F.. Data from: Exploring the effect of vibrotactile feedback through the floor on social presence in an immersive virtual environment. (2017) Available online at:http://pku.summon.serialssolutions.com/2.0.0/link/0/eLvHCXMwlV1LT8MwDI42TpwGbIi38gPolqRd0pzRJoRAmhBw4DLl4UjTpnaatv1-nLaUh7hwalUlsZQ0sT_Hn01IKoYs-XUm8NQ7x0BLQA2Xu8Clt5kNQQidGcsilfj9KX-cielk_NAhty01BgCqWDQYxtfqat-Xbhc9ZyOVa1Ruqku6Suc1davFWvhv6SaTD2d69PYco7jUsOnyo5RKpUmmvf8JPSKDL0oenbXK5ph0oDghvc-aDLTZon2yb4PqKJp2t A7XoGWge8TF5TbSGFZAAw5jjVvSpkxP1TasynJDy4LWfnS6rphJKHdR UFPQReXixsMRh9pE1gn9RpIbkNfp5OXuPmlqKySOp3KbKKs0YivInPA pcyhTSuWB49NmY8NBcDDWji0wKQy3aHhkJmQO7TdQ3or0lBwUZQFnhH pjNXNjxXyKcAtRqXQ6dk1xR5sg_Tnpx2mcr-v0GfNmBi_-_nxJDuNK1V6OK3Kw3ezgmnTXy91NtcIfz9CwiQ

[ref29] Li Y. Zheng W. (2021). Emotion recognition and regulation based on stacked sparse auto-encoder network and personalized reconfigurable music. Mathematics 9:593. doi: 10.3390/math9060593

[ref30] Litleskare S. MacIntyre T. E. Calogiuri G. (2020). Enable, reconnect and augment: a new ERA of virtual nature research and application. Int. J. Environ. Res. Public Health 17:1738. doi: 10.3390/ijerph17051738, PMID: 32155911 PMC7084893

[ref31] Maneuvrier A. Decker L. M. Ceyte H. Fleury P. Renaud P. (2020). Presence promotes performance on a virtual spatial cognition task: impact of human factors on virtual reality assessment. Front. Virtual Real. 1:571713. doi: 10.3389/frvir.2020.571713

[ref32] Martin D. Malpica S. Gutierrez D. Masia B. Serrano A. (2021). Multimodality in VR: a survey. 1:1. doi: 10.48550/arXiv.2101.07906

[ref33] Mathôt S. (2018). Pupillometry: psychology, physiology, and function. J. Cogn. 1:16. doi: 10.5334/joc.18, PMID: 31517190 PMC6634360

[ref34] Mauersberger H. Blaison C. Hess U. (2025). Task-irrelevant emotional expressions are not mimicked, but may modulate the mimicry of task-relevant emotional expressions. Front. Psychol. 15:1491832. doi: 10.3389/fpsyg.2024.1491832, PMID: 39839930 PMC11748183

[ref35] Meehan M. Insko B. Whitton M. Brooks F. P. Data from: Physiological measures of presence in stressful virtual environments. (2002) Available online at:https://go.exlibris.link/7vHlw2Qj 10.1007/s10484-005-6381-3 16167189

[ref36] Mobbs D. Yu R. Rowe J. B. Eich H. FeldmanHall O. Dalgleish T. (2010). Neural activity associated with monitoring the oscillating threat value of a tarantula. Proc. Natl. Acad. Sci. USA 107, 20582–20586. doi: 10.1073/pnas.1009076107, PMID: 21059963 PMC2996708

[ref37] Nayak S. Kuo C. Tsai A. C. (2019). Mid-frontal theta modulates response inhibition and decision making processes in emotional contexts. Brain Sci. 9:271. doi: 10.3390/brainsci9100271, PMID: 31614456 PMC6826545

[ref38] Okon-Singer H. Hendler T. Pessoa L. Shackman A. J. (2015). The neurobiology of emotion-cognition interactions: fundamental questions and strategies for future research. Front. Hum. Neurosci. 9:58. doi: 10.3389/fnhum.2015.00058, PMID: 25774129 PMC4344113

[ref39] Oliva M. Anikin A. (2018). Pupil dilation reflects the time course of emotion recognition in human vocalizations. Sci. Rep. 8:4871. doi: 10.1038/s41598-018-23265-x, PMID: 29559673 PMC5861097

[ref40] Ortuño-Sierra J. Santarén-Rosell M. de Albéniz A. P. Fonseca-Pedrero E. (2015). Dimensional structure of the Spanish version of the positive and negative affect schedule (PANAS) in adolescents and young adults. Psychol. Assess. 27, e1–e9. doi: 10.1037/pas0000107, PMID: 26029941

[ref41] Ozawa S. (2021). Emotions induced by recalling memories about interpersonal stress. Front. Psychol. 12:618676. doi: 10.3389/fpsyg.2021.618676, PMID: 33897528 PMC8062919

[ref42] Page S. Coxon M. (2016). Virtual reality exposure therapy for anxiety disorders: small samples and no controls? Front. Psychol. 7:326. doi: 10.3389/fpsyg.2016.00326, PMID: 27014137 PMC4786550

[ref43] Patidar V. (2024). A family of robust chaotic S-unimodal maps based on the Gaussian function. Front. Phys. 12:1328895. doi: 10.3389/fphy.2024.1328895

[ref44] Pei Y. Zhao S. Xie L. Luo Z. Zhou D. Ma C.. (2025). Identifying stable EEG patterns in manipulation task for negative emotion recognition. IEEE Trans. Affect. Comput. 1, 1–15. doi: 10.1109/TAFFC.2025.3551330, PMID: 40567712

[ref45] Peleskova S. Polak J. Janovcova M. Chomik A. Sedlackova K. Frynta D.. (2023). Human emotional evaluation of ancestral and modern threats: fear, disgust, and anger. Front. Psychol. 14:1321053. doi: 10.3389/fpsyg.2023.1321053, PMID: 38239483 PMC10794497

[ref46] Pessoa L. (2008). On the relationship between emotion and cognition. Nat. Rev. Neurosci. 9, 148–158. doi: 10.1038/nrn2317, PMID: 18209732

[ref47] Pessoa L. (2009). How do emotion and motivation direct executive control? Trends Cogn. Sci. 13, 160–166. doi: 10.1016/j.tics.2009.01.006, PMID: 19285913 PMC2773442

[ref48] Putkinen V. Nazari-Farsani S. Seppala K. Karjalainen T. Sun L. Karlsson H. K.. (2021). Decoding music-evoked emotions in the auditory and motor cortex. Cereb. Cortex 31, 2549–2560. doi: 10.1093/cercor/bhaa373, PMID: 33367590

[ref49] Raschle N. M. Fehlbaum L. V. Menks W. M. Euler F. Sterzer P. Stadler C. (2017). Investigating the neural correlates of emotion-cognition interaction using an affective Stroop task. Front. Psychol. 8:1489. doi: 10.3389/fpsyg.2017.01489, PMID: 28919871 PMC5585191

[ref50] Reisenzein R. (2019). Cognition and emotion: a plea for theory. Cogn. Emot. 33, 109–118. doi: 10.1080/02699931.2019.1568968, PMID: 30654695

[ref51] Reynolds G. Askew C. (2019). Effects of vicarious disgust learning on the development of fear, disgust, and attentional biases in children. Emotion 19, 1268–1283. doi: 10.1037/emo0000511, PMID: 30265075

[ref52] Rohr M. Degner J. Wentura D. (2022). Emotion misattribution from complex scene pictures: evidence for affective processing beyond valence. Emotion 22, 1208–1223. doi: 10.1037/emo0000901, PMID: 33180529

[ref53] Russo S. Tibermacine I. E. Tibermacine A. Chebana D. Nahili A. Starczewscki J.. (2024). Analyzing EEG patterns in young adults exposed to different acrophobia levels: a VR study. Front. Hum. Neurosci. 18:1348154. doi: 10.3389/fnhum.2024.1348154, PMID: 38770396 PMC11102978

[ref54] Schuetz I. Fiehler K. (2022). Eye tracking in virtual reality: Vive pro eye spatial accuracy, precision, and calibration reliability. J. Eye Mov. Res. 15:10.16910. doi: 10.16910/jemr.15.3.3, PMID: 37125009 PMC10136368

[ref55] Seeley W. W. Menon V. Schatzberg A. F. Keller J. Glover G. H. Kenna H.. (2007). Dissociable intrinsic connectivity networks for salience processing and executive control. J. Neurosci. 27, 2349–2356. doi: 10.1523/JNEUROSCI.5587-06.2007, PMID: 17329432 PMC2680293

[ref56] Senderecka M. (2018). Emotional enhancement of error detection—the role of perceptual processing and inhibition monitoring in failed auditory stop trials. Cogn. Affect. Behav. Neurosci. 18, 1–20. doi: 10.3758/s13415-017-0546-4, PMID: 29076064 PMC5823965

[ref57] Shanmughapriya M. Sanchana R. Josephine R. (2024). A survey on multimodal sensory interaction in augmented reality and virtual reality. EPRA Int. J. Res. Develop. 3, 2455–7838. doi: 10.36713/epra16270

[ref58] Solms M. Jurist E. L. (2017). Review of the cognitive-emotional brain: from interactions to integration and the feeling body: affective neuroscience meets the enactive mind. Psychoanal. Psychol. 34, 122–130. doi: 10.1037/pap0000088

[ref59] Sussman T. J. Heller W. Miller G. A. Mohanty A. (2013). Emotional distractors can enhance attention. Psychol. Sci. 24, 2322–2328. doi: 10.1177/0956797613492774, PMID: 24058065 PMC4408991

[ref60] Sutherland M. R. Mather M. (2018). Arousal (but not valence) amplifies the impact of salience. Cogn. Emot. 32, 616–622. doi: 10.1080/02699931.2017.1330189, PMID: 28566006 PMC5786489

[ref61] Teachman B. A. Stefanucci J. K. Clerkin E. M. Cody M. W. Proffitt D. R. Phelps E. A. (2008). A new mode of fear expression: perceptual bias in height fear. Emotion 8, 296–301. doi: 10.1037/1528-3542.8.2.296 18410203 PMC3182306

[ref62] Teggi R. Comacchio F. Fornasari F. Mira E. (2019). Height intolerance between physiological mechanisms and psychological distress: a review of literature and our experience. Acta Otorhinolaryngol. Ital. 39, 263–268. doi: 10.14639/0392-100X-2190, PMID: 31501618 PMC6734202

[ref63] Venkatesan R. K. Banakou D. Slater M. Manivannan M. (2023). Haptic feedback in a virtual crowd scenario improves the emotional response. Front. Virtual Real. 4:1242587. doi: 10.3389/frvir.2023.1242587

[ref64] Vieillard S. Ronat L. Baccarani A. Schaal B. Baudouin J. Brochard R. (2021). Age differences in olfactory affective responses: evidence for a positivity effect and an emotional dedifferentiation. Aging Neuropsychol. Cogn. 28, 570–583. doi: 10.1080/13825585.2020.1799926, PMID: 32787505

[ref65] Villani D. Carissoli C. Triberti S. Marchetti A. Gilli G. Riva G. (2018). Videogames for emotion regulation: a systematic review. Games Health J. 7, 85–99. doi: 10.1089/g4h.2017.0108, PMID: 29424555

[ref66] Vrontos A. Nitsch V. Brandl C. (2024). Electrical muscle stimulation for kinesthetic feedback in AR/VR: a systematic literature review. Multimodal Technol. Interact. 8:7. doi: 10.3390/mti8020007

[ref67] Wagener N. Ackermann A. Savino G. Dänekas B. Niess J. Schöning J. Data from: Influence of passive haptic and auditory feedback on presence and mindfulness in virtual reality environments. (2022) Available online at: https://go.exlibris.link/GlmyV1zp

[ref68] Wan P. Jing X. Lu S. Yan L. (2023). Impact of temperament types and anger intensity on drivers' EEG power spectrum and sample entropy: an on-road evaluation toward road rage warning. Tehnicki vjesnik - Technical Gazette 30, 1055–1067. doi: 10.17559/TV-20221021054632

[ref69] Watson D. Clark L. A. Tellegen A. (1988). Development and validation of brief measures of positive and negative affect: the PANAS scales. J. Pers. Soc. Psychol. 54, 1063–1070. doi: 10.1037/0022-3514.54.6.1063 3397865

[ref70] Wei B. A. Yang B. Zhang W. Liu P. Fu H. Lv Z.. (2024). Construction site hazard identification and worker adverse reaction monitoring using electroencephalograms: a review. Buildings 14:180. doi: 10.3390/buildings14010180

[ref71] Xie Z. Pan J. Li S. Ren J. Qian S. Ye Y.. (2022). Musical emotions recognition using entropy features and channel optimization based on EEG. Entropy 24:1735. doi: 10.3390/e24121735, PMID: 36554139 PMC9777832

[ref72] Yang J. Tang M. Cong L. Sun J. Guo D. Zhang T.. (2023). Development and validation of an assessment index for quantifying cognitive task load in pilots under simulated flight conditions using heart rate variability and principal component analysis. Ergonomics 67:1. doi: 10.1080/00140139.2023.2229075, PMID: 37365918

[ref73] Zhang J. Wu C. Yuan Z. Meng Y. (2019). Differentiating emotion-label words and emotion-laden words in emotion conflict: an ERP study. Exp. Brain Res. 237, 2423–2430. doi: 10.1007/s00221-019-05600-4, PMID: 31302735

[ref74] Zhang X. Yu H. W. Barrett L. F. (2014). How does this make you feel? A comparison of four affect induction procedures. Front. Psychol. 5:689. doi: 10.3389/fpsyg.2014.00689, PMID: 25071659 PMC4086046

