# Supplementary material for: Haptic feedback as affective amplifier: enhanced fear perception affects cognitive performance and avoidance actions in VR height exposure
Source: Front Psychol. 2025 Jul 23;16:1560157. doi: 10.3389/fpsyg.2025.1560157 (PMC12325401; doi:10.3389/fpsyg.2025.1560157)
Supplement: Supplementary file 1 [file Supplementary_file_1.DOCX]

Table S1 The experimental randomization matrix design.

| Participant | Session 1  (Week 1) | Session 2  (Week 2) | Session 3  (Week 3) | Session 4  (Week 4) |
| --- | --- | --- | --- | --- |
| Participant 1-5  (group1) | Neutrality | Ground | Stationary | Shaking |
| Participant 6-10  (group2) | Ground | Stationary | Shaking | Neutrality |
| Participant 11-15  (group3) | Stationary | Shaking | Neutrality | Ground |
| Participant 16-20  (group4) | Shaking | Neutrality | Ground | Stationary |

Table S2 Results of the interaction effect between fear score measurement stage and induced condition

| Parameter | SS | df | MS | F | *p* | η² |
| --- | --- | --- | --- | --- | --- | --- |
| Measurement stage | 61.256 | 1 | 61.256 | 5.205* | .034 | .215 |
| Induced condition | 167.969 | 3 | 55.990 | 16.106*** | <.001 | .459 |
| Measurement stage*Induced condition | 37.119 | 3 | 12.373 | 3.683* | .017 | .162 |

The significance levels are marked as follows:*p<0.05, ***p<0.001.

| Table S3 Simple effect analysis in the measurement stage of fear scores | | | | | | | | | |
| --- | --- | --- | --- | --- | --- | --- | --- | --- | --- |
| Comparison | | Mean | Std. Deviation | Std. Error Mean | 95% Confidence Interval of the Difference | | t | df | *p* |
|  |  |  |  |  | Lower | Upper |  |  |  |
| Pair 1 neutrality | pre - post | .40000 | 2.18608 | .48882 | -.62312 | 1.42312 | .818 | 19 | .423 |
| Pair 2 ground | pre - post | -.75000 | 3.32257 | .74295 | -2.30501 | .80501 | -1.009 | 19 | .325 |
| Pair 3 stationary | pre - post | -1.30000 | 3.26222 | .72946 | -2.82677 | .22677 | -1.782 | 19 | .091 |
| Pair 4  shaking | pre - post | -2.75000 | 4.29044 | .95937 | -4.75799 | -.74201 | -2.866* | 19 | .010 |

The significance levels are marked as follows:*p<0.05.

Table S4 Simple effect analysis in the induced condition of fear scores

| Source | SS | df | MS | F | p | η² |
| --- | --- | --- | --- | --- | --- | --- |
| Pre | 10.738 | 3 | 3.579 | 2.193 | .120 | .103 |
| Post | 179.638 | 3 | 59.879 | 13.046*** | <.001 | .407 |

The significance levels are marked as follows:***p<0.001.

Table S5 The post-hoc pairwise comparison results of eye movement

| Source | (I) Factor | (J) Factor | Mean Difference (I-J) | Std. Error | p | 95% Confidence Interval for Difference | |
| --- | --- | --- | --- | --- | --- | --- | --- |
|  |  |  |  |  |  | Lower Bound | Upper Bound |
| Pre | neutrality | ground | -.850 | .449 | .441 | -2.171 | .471 |
|  |  | stationary | -1.100 | .458 | .161 | -2.449 | .249 |
|  |  | shaking | -1.550 | .596 | .105 | -3.304 | .204 |
|  | ground | neutrality | .850 | .449 | .441 | -.471 | 2.171 |
|  |  | stationary | -.250 | .362 | 1.000 | -1.315 | .815 |
|  |  | shaking | -.700 | .417 | .659 | -1.929 | .529 |
|  | stationary | neutrality | 1.100 | .458 | .161 | -.249 | 2.449 |
|  |  | ground | .250 | .362 | 1.000 | -.815 | 1.315 |
|  |  | shaking | -.450 | .526 | 1.000 | -1.997 | 1.097 |
|  | shaking | neutrality | 1.550 | .596 | .105 | -.204 | 3.304 |
|  |  | ground | .700 | .417 | .659 | -.529 | 1.929 |
|  |  | stationary | .450 | .526 | 1.000 | -1.097 | 1.997 |
| Post | neutrality | ground | -1.450 | .550 | .098 | -3.069 | .169 |
|  |  | stationary | -2.250** | .528 | .003 | -3.803 | -.697 |
|  |  | shaking | -4.150*** | .776 | <.001 | -6.433 | -1.867 |
|  | ground | neutrality | 1.450 | .550 | .098 | -.169 | 3.069 |
|  |  | stationary | -.800 | .651 | 1.000 | -2.717 | 1.117 |
|  |  | shaking | -2.700* | .758 | .012 | -4.931 | -.469 |
|  | stationary | neutrality | 2.250** | .528 | .003 | .697 | 3.803 |
|  |  | ground | .800 | .651 | 1.000 | -1.117 | 2.717 |
|  |  | shaking | -1.900 | .757 | .128 | -4.129 | .329 |
|  | shaking | neutrality | 4.150*** | .776 | <.001 | 1.867 | 6.433 |
|  |  | ground | 2.700* | .758 | .012 | .469 | 4.931 |
|  |  | stationary | 1.900 | .757 | .128 | -.329 | 4.129 |

Adjustment for multiple comparisons: Bonferroni. The significance levels are marked as follows:*p<0.05, ***p*<0.01, ***p<0.001.

Table S6 The post-hoc pairwise comparison results of eye movement

| Parameter | (I) Factor | (J) Factor | Mean Difference (I-J) | Std. Error | p | 95% Confidence Interval for Difference | |
| --- | --- | --- | --- | --- | --- | --- | --- |
|  |  |  |  |  |  | Lower Bound | Upper Bound |
| Left eye saccade amplitude | neutrality | ground | .301 | .689 | 1.000 | -1.726 | 2.329 |
|  |  | stationary | -2.235 | .781 | .060 | -4.536 | .066 |
|  |  | shaking | -1.467 | 1.315 | 1.000 | -5.338 | 2.404 |
|  | ground | neutrality | -.301 | .689 | 1.000 | -2.329 | 1.726 |
|  |  | stationary | -2.536* | .664 | .007 | -4.492 | -.580 |
|  |  | shaking | -1.768 | 1.072 | .693 | -4.924 | 1.388 |
|  | stationary | neutrality | 2.235 | .781 | .060 | -.066 | 4.536 |
|  |  | ground | 2.536* | .664 | .007 | .580 | 4.492 |
|  |  | shaking | .768 | 1.230 | 1.000 | -2.852 | 4.388 |
|  | shaking | neutrality | 1.467 | 1.315 | 1.000 | -2.404 | 5.338 |
|  |  | ground | 1.768 | 1.072 | .693 | -1.388 | 4.924 |
|  |  | stationary | -.768 | 1.230 | 1.000 | -4.388 | 2.852 |
| Right eye saccade amplitude | neutrality | ground | .001 | .808 | 1.000 | -2.378 | 2.380 |
|  |  | stationary | -1.019 | 1.234 | 1.000 | -4.650 | 2.613 |
|  |  | shaking | .397 | 1.299 | 1.000 | -3.428 | 4.222 |
|  | ground | neutrality | -.001 | .808 | 1.000 | -2.380 | 2.378 |
|  |  | stationary | -1.020 | 1.045 | 1.000 | -4.098 | 2.058 |
|  |  | shaking | .395 | 1.031 | 1.000 | -2.641 | 3.432 |
|  | stationary | neutrality | 1.019 | 1.234 | 1.000 | -2.613 | 4.650 |
|  |  | ground | 1.020 | 1.045 | 1.000 | -2.058 | 4.098 |
|  |  | shaking | 1.416 | 1.206 | 1.000 | -2.134 | 4.965 |
|  | shaking | neutrality | -.397 | 1.299 | 1.000 | -4.222 | 3.428 |
|  |  | ground | -.395 | 1.031 | 1.000 | -3.432 | 2.641 |
|  |  | stationary | -1.416 | 1.206 | 1.000 | -4.965 | 2.134 |
| Left eye pupil diameter | neutrality | ground | -.068 | .158 | 1.000 | -.533 | .397 |
|  |  | stationary | -.221 | .115 | .422 | -.561 | .119 |
|  |  | shaking | -.526*** | .118 | .002 | -.874 | -.178 |
|  | ground | neutrality | .068 | .158 | 1.000 | -.397 | .533 |
|  |  | stationary | -.153 | .162 | 1.000 | -.631 | .324 |
|  |  | shaking | -.458* | .155 | .048 | -.913 | -.003 |
|  | stationary | neutrality | .221 | .115 | .422 | -.119 | .561 |
|  |  | ground | .153 | .162 | 1.000 | -.324 | .631 |
|  |  | shaking | -.305* | .090 | .018 | -.569 | -.041 |
|  | shaking | neutrality | .526*** | .118 | .002 | .178 | .874 |
|  |  | ground | .458* | .155 | .048 | .003 | .913 |
|  |  | stationary | .305* | .090 | .018 | .041 | .569 |
| Right eye pupil diameter | neutrality | ground | -.064 | .149 | 1.000 | -.504 | .376 |
|  |  | stationary | -.074 | .140 | 1.000 | -.485 | .337 |
|  |  | shaking | -.423* | .126 | .019 | -.793 | -.053 |
|  | ground | neutrality | .064 | .149 | 1.000 | -.376 | .504 |
|  |  | stationary | -.010 | .199 | 1.000 | -.594 | .575 |
|  |  | shaking | -.359 | .180 | .363 | -.889 | .171 |
|  | stationary | neutrality | .074 | .140 | 1.000 | -.337 | .485 |
|  |  | ground | .010 | .199 | 1.000 | -.575 | .594 |
|  |  | shaking | -.349* | .101 | .016 | -.647 | -.052 |
|  | shaking | neutrality | .423* | .126 | .019 | .053 | .793 |
|  |  | ground | .359 | .180 | .363 | -.171 | .889 |
|  |  | stationary | .349* | .101 | .016 | .052 | .647 |
| Left eye openness | neutrality | ground | .006 | .012 | 1.000 | -.029 | .042 |
|  |  | stationary | -.025 | .019 | 1.000 | -.081 | .030 |
|  |  | shaking | -.012 | .022 | 1.000 | -.078 | .053 |
|  | ground | neutrality | -.006 | .012 | 1.000 | -.042 | .029 |
|  |  | stationary | -.032 | .022 | 1.000 | -.097 | .034 |
|  |  | shaking | -.018 | .025 | 1.000 | -.093 | .057 |
|  | stationary | neutrality | .025 | .019 | 1.000 | -.030 | .081 |
|  |  | ground | .032 | .022 | 1.000 | -.034 | .097 |
|  |  | shaking | .013 | .020 | 1.000 | -.045 | .071 |
|  | shaking | neutrality | .012 | .022 | 1.000 | -.053 | .078 |
|  |  | ground | .018 | .025 | 1.000 | -.057 | .093 |
|  |  | stationary | -.013 | .020 | 1.000 | -.071 | .045 |
| Right eye openness | neutrality | ground | -.033 | .019 | .659 | -.090 | .025 |
|  |  | stationary | -.018 | .021 | 1.000 | -.079 | .044 |
|  |  | shaking | -.042 | .017 | .148 | -.094 | .009 |
|  | ground | neutrality | .033 | .019 | .659 | -.025 | .090 |
|  |  | stationary | .015 | .019 | 1.000 | -.040 | .070 |
|  |  | shaking | -.010 | .011 | 1.000 | -.043 | .023 |
|  | stationary | neutrality | .018 | .021 | 1.000 | -.044 | .079 |
|  |  | ground | -.015 | .019 | 1.000 | -.070 | .040 |
|  |  | shaking | -.025 | .020 | 1.000 | -.085 | .035 |
|  | shaking | neutrality | .042 | .017 | .148 | -.009 | .094 |
|  |  | ground | .010 | .011 | 1.000 | -.023 | .043 |
|  |  | stationary | .025 | .020 | 1.000 | -.035 | .085 |

Adjustment for multiple comparisons: Bonferroni. The significance levels are marked as follows:*p<0.05, ***p<0.001.
